# Disagreements in risk of bias assessment for randomized controlled trials in hypertension-related Cochrane reviews

**DOI:** 10.1186/s13063-024-08145-2

**Published:** 2024-06-21

**Authors:** Yi Yao, Jing Shen, Jianzhao Luo, Nian Li, Xiaoyang Liao, Yonggang Zhang

**Affiliations:** 1 General Practice Ward/International Medical Center Ward, General Practice Medical Center, West China Hospital, Sichuan University, Chengdu, 610041 China; 2 Department of Medical Administration, West China Hospital, Sichuan University, Chengdu, 610041 China; 3 Department of Periodical Press and National Clinical Research Center for Geriatrics, West China Hospital, Sichuan University, Chengdu, 610041 China

**Keywords:** Systematic review, Randomized controlled trial, Risk of bias, Cochrane

## Abstract

**Background:**

The inter-reviewer reliability of the risk of bias (RoB) assessment lacked agreement in previous studies. It is important to analyse these disagreements to improve the repeatability of RoB assessment**.** The objective of the study was to evaluate the frequency and reasons for disagreements in RoB assessments for randomised controlled trials (RCTs) that were included in multiple Cochrane reviews in the field of hypertension.

**Methods:**

A cross-sectional study was employed. We retrieved any RCTs that had been included in multiple Cochrane reviews in the field of hypertension from ARCHIE. The results of the RoB assessments were extracted, and the distributions of agreements and possible reasons for disagreement were analyzed.

**Results:**

Twenty-six Cochrane reviews were included in this study. A total of 78 RCTs appeared in more than one Cochrane review. The level of agreement ranged from domain to domain. “Blinding of outcome assessment” showed a reasonably high level of agreement (94.9%), while “incomplete outcome data”, “selective outcome reporting” and “other sources of bias” showed moderate levels of agreement (74.6%, 79.2% and 75.6%, respectively). However, the domains of “allocation concealment”, “random sequence generation” and “blinding of participants and personnel” showed low levels of agreement (24.4%, 23.5%, and 47.4%, respectively). In the domains of “allocation concealment” and “blinding of participants and personnel”, the agreement group had higher proportion of publication year ≤ 1996 than the disagreement group (*P* = 0.008 and *P* < 0.001, respectively). In the “blinding of participants and personnel”, the impact factor was higher in the agreement group (*P* < 0.001). By analyzing the support text, we found that the most likely reason for disagreement was extracting different information from the same RCT.

**Conclusion:**

For Cochrane reviews in the field of hypertension using the 2011 version of the RoB tool, there was a large disagreement in the RoB assessment. It is suggested that the results of RoB assessments in systematic reviews that used the 2011 version of the RoB tool need to be interpreted with caution. More accurate information from RCTs needs to be collected when we synthesize clinical evidence.

**Supplementary Information:**

The online version contains supplementary material available at 10.1186/s13063-024-08145-2.

## Background

Hypertension is a major health problem worldwide, causing 17.9 million deaths each year [1]. The prevention and treatment of hypertension is thus an important issue [2]. Systematic reviews involving randomized controlled trials (RCTs) provide credible evidence for the prevention and treatment of hypertension and guide health care and policy decision-making [3]. However, bias in the RCTs could bias the estimates of interventions. Previous studies showed that the summary results of low-quality RCTs might exaggerate the treatment effect [4–6]. Therefore, evaluating the risk of bias (RoB) becomes crucial in determining the quality of RCTs [7, 8].

In 2008, the Cochrane Collaboration released a tool to assess the RoB for RCTs [9, 10]. This tool was developed through an extensive process of improving other tools for quality assessment and was updated in 2011 [9]. It includes assessments and comments for 7 domains of bias: “random sequence generation”, “allocation concealment”, “blinding of participants and personnel”, “blinding of outcome assessment”, “incomplete outcome data”, “selective outcome reporting” and “other sources of bias”. For each domain, the reviewers assessed it as either high risk, low risk or unclear risk. It was recommended that RoB should be assessed independently by two reviewers, and that disagreements should be resolved by consensus or by a third reviewer [11]. However, different reviewers might carry out different assessments, leading to unsatisfactory inter-reviewer reliability in RoB assessments [12, 13]. The disagreement might have a negative impact on the interpretation of evidence from systematic reviews, consequently impacting decision-making processes and the quality of healthcare. Bertizzolo et al. [14] included 1604 RCTs in more than one Cochrane review published between March 2011 and September 2014 and reported that RoB assessment varied significantly among different groups and agreement ranged from 57% for “incomplete outcome data” to 81% for “random sequence generation”, and the agreement of “blinding of participants and personnel” and “blinding of outcome assessment” was moderate level (72% and 67%, respectively). Disagreement in RoB assessments varied across different research fields. Jordan et al. found [15] that there was a reasonably high level of agreement in the domains of “random sequence generation” and “incomplete outcome data” (71% and 79%, respectively) in the field of subfertility; for the domain of blinding, agreement was reached in only 35% of cases.

In the field of hypertension, there was no such a study has assessed disagreements in the RoB for RCTs. Thus, the current study was performed. In this study, we compared the RoB assessment across multiple Cochrane reviews, rather than just across two reviews, to look at multiple variabilities in RoB assessments of the same trial. The characteristics of the included RCTs and Cochrane reviews and the support text of the RoB assessment were analyzed to find the possible reasons for the disagreement.

## Methods

### Study design

A cross-sectional design was employed. Any RCT that had been included in more than one Cochrane review of hypertension was identified. For each domain of the RoB tool, the level of agreement and disagreement between different reviews was assessed.

### Data sources

We exported all Cochrane reviews in the hypertension group from ARCHIE (https://archie.cochrane.org/resources.jsp) in the Cochrane Library between June 13, 2008, and December 31, 2020. The extracted information includes details such as the title, publication status, review status, review type and review CD number.

### Selection of eligible Cochrane reviews and extraction of data

The exclusion criteria for reviews were as follows: 1) revocatory publications; 2) inactive Cochrane reviews; 3) intervention protocols; and 4) Cochrane reviews without RCTs. Information about the included reviews was extracted, including the country of the first author, year of publication, number of participants, and number of RCTs.

### Selection of eligible RCTs

We compiled a list of all RCTs for the included Cochrane reviews using Excel software. The RCTs were represented by the “first author’s last name & year of publication”, according to the Cochrane handbook [11]. Excel’s sorting function was used to find the same and similar RCTs and determine whether they were included in one or more reviews. RCTs with the same reference were considered the same RCT in different reviews. The process was carried out independently by two authors, and disagreements were resolved by discussion. We excluded 1) RCTs with the same/similar “author name & year” from one Cochrane review (because an RCT may appear multiple times in the same Cochrane review. This situation can be seen in “Effect of cocoa on blood pressure” [16]); 2) RCTs with a similar “author last name & year” (e.g., ACCORD 2010 and ACCORD BP 2010), but corresponding to different references; 3) RCTs with the same “last author name & year” had different references (e.g., AASK 2002 and AASK 2002); and 4) RCTs in which the reviewers did not use the 2011 version of the RoB tool. The same RCT might have been counted multiple times and had multiple results of RoB assessments when an RCT was included in several Cochrane reviews. Therefore, we defined cases in which one RCT was included by several Cochrane reviews as a group. For example, when an RCT was included in 3 Cochrane reviews, we defined it as one group. In this group, the RCT was counted 3 times during the data collection and there were 3 results of RoB assessments. We also extracted data for these RCTs from the Cochrane reviews and Web of Science database, This data extraction encompassed information such as the publication journal, impact factor and year of publication. The impact factors were from the Journal Citation Report (2019). When one RCT corresponded to multiple references in reviews, the data were extracted from the most recent publication.

### Extraction results of the RoB assessment

For RCTs included in more than one Cochrane review, we extracted the results of the RoB assessment and outcomes for the RCT from “Characteristics of included studies” in Cochrane reviews. For example, if one RCT was reported in 5 reviews, we extracted the results of the RoB assessment and outcomes from the 5 reviews. We also extracted the “support text” for the RoB assessment in each Cochrane review.

### Comparison of RoB assessments among Cochrane reviews

For RCTs included in more than one Cochrane review, the results of the RoB assessment among different reviews were compared. First, we evaluated the level of agreement or disagreement of the overall RoB assessment at the RCT level. The overall assessment of the RoB at the RCT level was determined using the following criteria: an RCT was considered to have an overall low RoB if all domains assessed were classified as low risk, an overall high RoB if any of the domains were classified as high risk, and an overall unclear RoB if the domains were classified as either low risk or unclear risk. Second, we evaluated the level of agreement and disagreement for each domain of the RoB tool. There were only 5 possibilities when one RCT was included in several Cochrane reviews: agreement, low *vs.* unclear, unclear *vs.* high, low *vs.* high and low *vs.* unclear *vs.* high. For example, there were theoretically 27 possibilities for an RCT included in 3 reviews. However, most of the possibilities were duplicates. In the case of “low risk *vs.* low risk *vs.* unclear risk”, we simplified it to “low risk *vs.* unclear risk”. Thus, we reduced 27 possibilities to 5. The percentage of 5 possibilities for each domain of RoB was calculated. According to the Cochrane handbook, the assessment of “blinding of participants and personnel”, “blinding of outcome assessment”, and “incomplete outcome data” was affected by the specific outcomes. However, when it comes to “blinding of participants and personnel” and “blinding of outcome assessment”, outcomes with similar RoB are usually assessed in groups rather than individually. Generally, all subjective outcomes were assessed separately from objective outcomes and each had an overall assessment result. In this study, if all outcomes within a group of reviews are objective outcomes or all are subjective outcomes, the RoB for blinding is similar, and result of assessment was not affected by the outcomes. The “incomplete outcome data” was still affected by outcomes, despite the subtle differences. For the domain of “incomplete outcome data”, we analyzed only RCTs that focused on the same outcomes in different reviews. Not all Cochrane reviews assessed all 7 RoB domains for each RCT, and the number of RoB assessments of RCTs varied depending on the considered domain.

### Analysis of the possible reasons for disagreement in the RoB assessment

The RCTs’ publication year and impact factor of the journal were compared between the agreement and disagreement groups of the RoB assessment. We divided the publication years into ≤ 1996 and > 1996 because the CONSORT statement was first published in 1996 [17, 18]. Continuous data are expressed as standard deviation (SD) if normally distributed or as median with interquartile range (IQR) if non-normally distributed. Enumeration data were described by frequencies and percentages. Continuous variables were analyzed based on the Wilcoxon rank sum test. The enumeration data were analysed using the Pearson Chi-squared test and Fisher's exact test. A *P* value of < 0.05 was considered to indicate statistical significance. The data were analysed by SPSS 23.0 software. The support text for all disagreements in the RoB assessment was manually evaluated. The possible reasons for disagreement were analysed through the differences in support text.

## Results

### Selection process

**Fig. 1:**
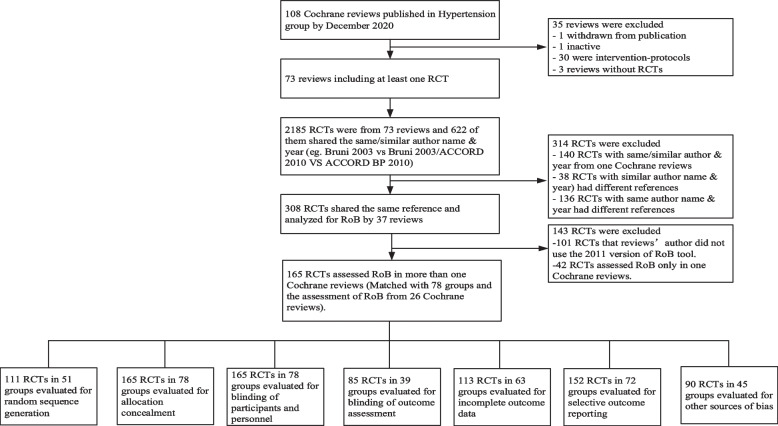
The selection process

We retrieved 108 hypertension-related Cochrane reviews up to December 31, 2020, of which 35 were excluded for the following reasons: 1 was withdrawn from publication, 1 was inactive, 30 were intervention-protocols, and 3 reviews were without RCTs. Seventy-three Cochrane reviews included at least one RCT, and 2185 RCTs were included in these reviews, of which 622 shared the same/similar “author name & year” (*e.g.,* Bruni 2003 *vs.* Bruni 2003/ACCORD 2010 *vs.* ACCORD BP 2010). We manually checked RCTs that shared the same reference in different reviews and excluded 314 RCTs for the following reasons: 140 RCTs with the same/similar “author name & year” from one Cochrane review, 38 RCTs with similar “author name & year” had different references, and 136 RCTs with same “author name & year” had different references. A total of 308 RCTs shared the same reference. Among the 308 RCTs, 101 RCTs that reviews’ authors did not use the 2011 version of the RoB tool. Forty-two RCTs were assessed the RoB in only one Cochrane review. A total of 165 RCTs from 26 Cochrane reviews were included and matched with 78 groups. Among the 165 RCTs, 111 RCTs in 51 groups were evaluated for “random sequence generation”. A total of 165 RCTs in 78 groups were evaluated for “allocation concealment”. All outcomes in our study were objective, so the RoB assessments of blinding were not affected by the outcomes, despite their subtle differences. A total of 165 RCTs in 78 groups were evaluated for “blinding of participants and personnel”. And 85 RCTs in 39 groups were evaluated for “blinding of outcome assessment”. A total of 161 RCTs in 76 groups were evaluated for “incomplete outcome data”. Twenty-six RCTs in 13 groups focused on different outcomes in different reviews. Therefore, only 113 RCTs in 63 groups were analysed for “incomplete outcome data”. A total of 152 RCTs in 72 groups were evaluated for “selective outcome reporting”. Ninety RCTs in 44 groups were evaluated for “other sources of bias”. Fig. 1 shows the selection process.

### Characteristics of the included Cochrane reviews

**Fig. 2:**
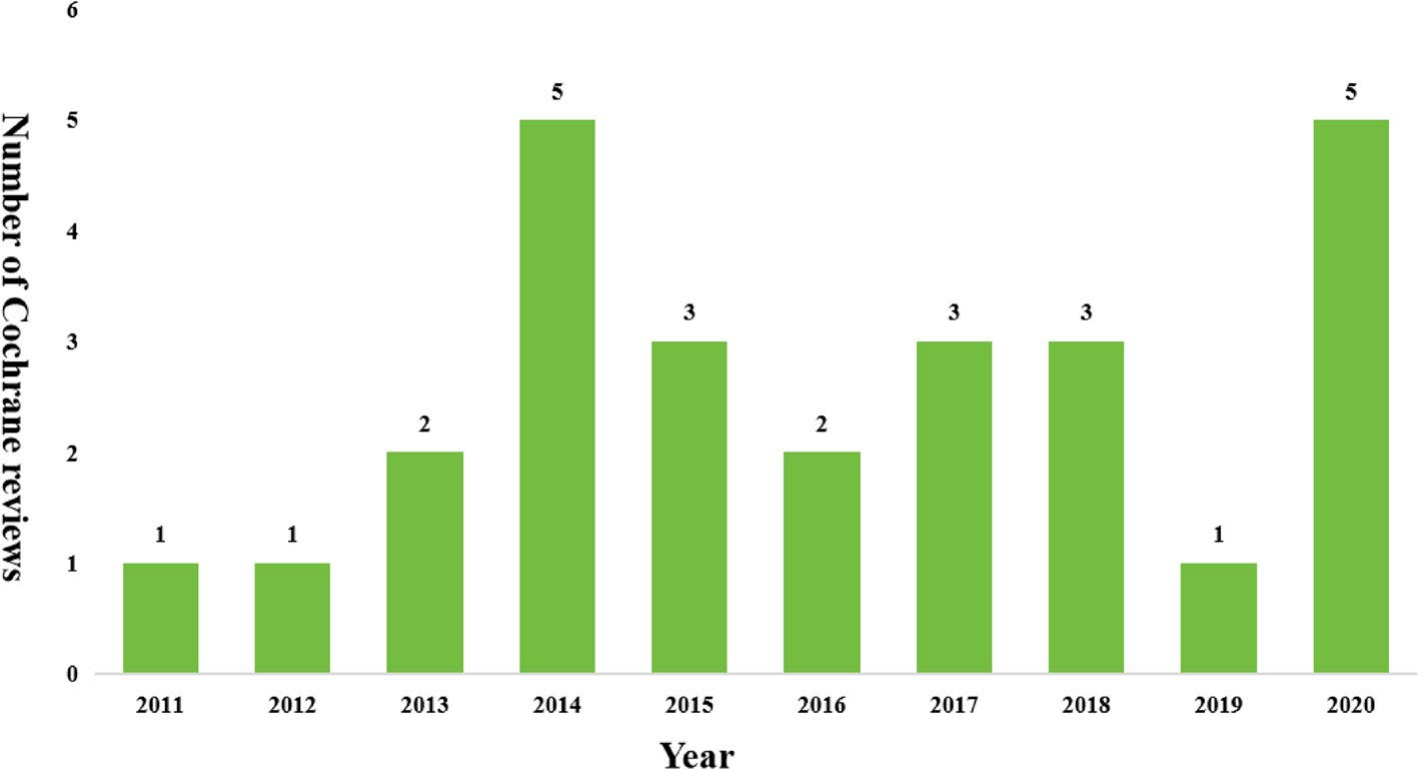
The publication trends of the included Cochrane reviews

The characteristics of the included Cochrane reviews are shown in Table 1. Of the 26 reviews included in this study, Canada produced the most reviews (61.5%), followed by the UK (7.6%), Costa Rica (7.6%) and China (2.7%). The median number of RCTs included in the Cochrane reviews was 16. The median number of participants in the Cochrane reviews was 11,789. The tool for assessing all RCTs in this study was the 2011 version of the RoB, not the RoB 2.0. The publication trends of the reviews are shown in Fig. 2.

**Table 1:** Characteristics of included the Cochrane reviews

Variables	Cochrane reviews
Country, *n* (%)	
Canada	16/26(61.5)
UK	2/26(7.6)
Costa Rica	2/26(7.6)
China	1/263.8)
Argentina	1/26(3.8)
Denmark	1/26(3.8)
France	1/26(3.8)
South Africa	1/26(3.8)
Spain	1/26(3.8)
Number of participants [M(IQR)]	11,789.0(2878.0,38,720.0)
Number of RCTs[M(IQR)]	16.0(9.8,51.5)

*M* median, *IQR* interquartile range

### Characteristics of the included RCTs

A total of 78 RCTs were included in the study. Most RCTs were published in the *Lancet* (11.5%), followed by the *Journal of Hypertension* (10.3%), *Hypertension* (7.7%), *BMJ- British Medical Journal* (7.7%), *American Journal of Cardiology* (3.8%), *Therapeutic Research* (2.6%), *JAMA* (2.6%), and *European Journal of Clinical Pharmacology* (2.6%). Details of the journals published by the included RCTs are shown in Supplementary Material 1. The median impact factor for journals was 4.2. Of the included RCTs, 92.3% were included in two reviews, 6.4% were included in three reviews, and 1.3% were included in four reviews. Most RCTs were from the USA (33.3%), followed by the UK (15.4%). In terms of research topics, 69.2% examined drug therapy, and 30.8% examined non-pharmacologic treatment (Table 2).

**Table 2:** Characteristics of RCTs in Cochrane reviews

Variables	RCT
Country [*n* (%)]	
USA	26(33.3)
UK	12(15.4)
Japan	8(10.3)
Italy	5(6.4)
Australia	3(3.8)
China	3(3.8)
Netherlands	3(3.8)
Sweden	3(3.8)
Germany	2(2.6)
Norway	2(2.6)
Other^**†**^	11(14.1)
Impact factor [M (IQR)]	4.2(2.6, 23.6)
Year, [*n* (%)]	
1970–1980	6 (7.7)
1981–1990	15 (19.2)
1991–2000	19 (24.4)
2001–2010	30 (38.5)
2011–2020	8(10.3)
Reviews corresponding to RCT [ *n* (%)]	
One RCT was included in 2 Cochrane reviews	72(92.3)
One RCT was included in 3 Cochrane reviews	5(6.4)
One RCT was included in 4 Cochrane reviews	1(1.3)
Journal [*n* (%)]	
Lancet	9(11.5)
Journal of Hypertension	8(10.3)
Hypertension	6(7.7)
BMJ- British Medical Journal	6(7.7)
American Journal of Cardiology	3(3.8)
Therapeutic Research	2(2.6)
JAMA	2(2.6)
European Journal of Clinical Pharmacology	2(2.6)
Current Medical Research & Opinion	2(2.6)
Clinical Therapeutics	2(2.6)
Others	36(46.2)
Research topic [*n*(%)]	
Drug therapy	54(69.2)
Nonpharmacologic therapy	24(30.8)

*M* median, *IQR* interquartile range

**†**Belgium, Canada, Denmark, Finland, France, Hungary, New Zealand, Poland, South Korea, Spain, Thailand

### Assessment of agreements and disagreements

#### Assessment of RCT level

**Fig. 3:**
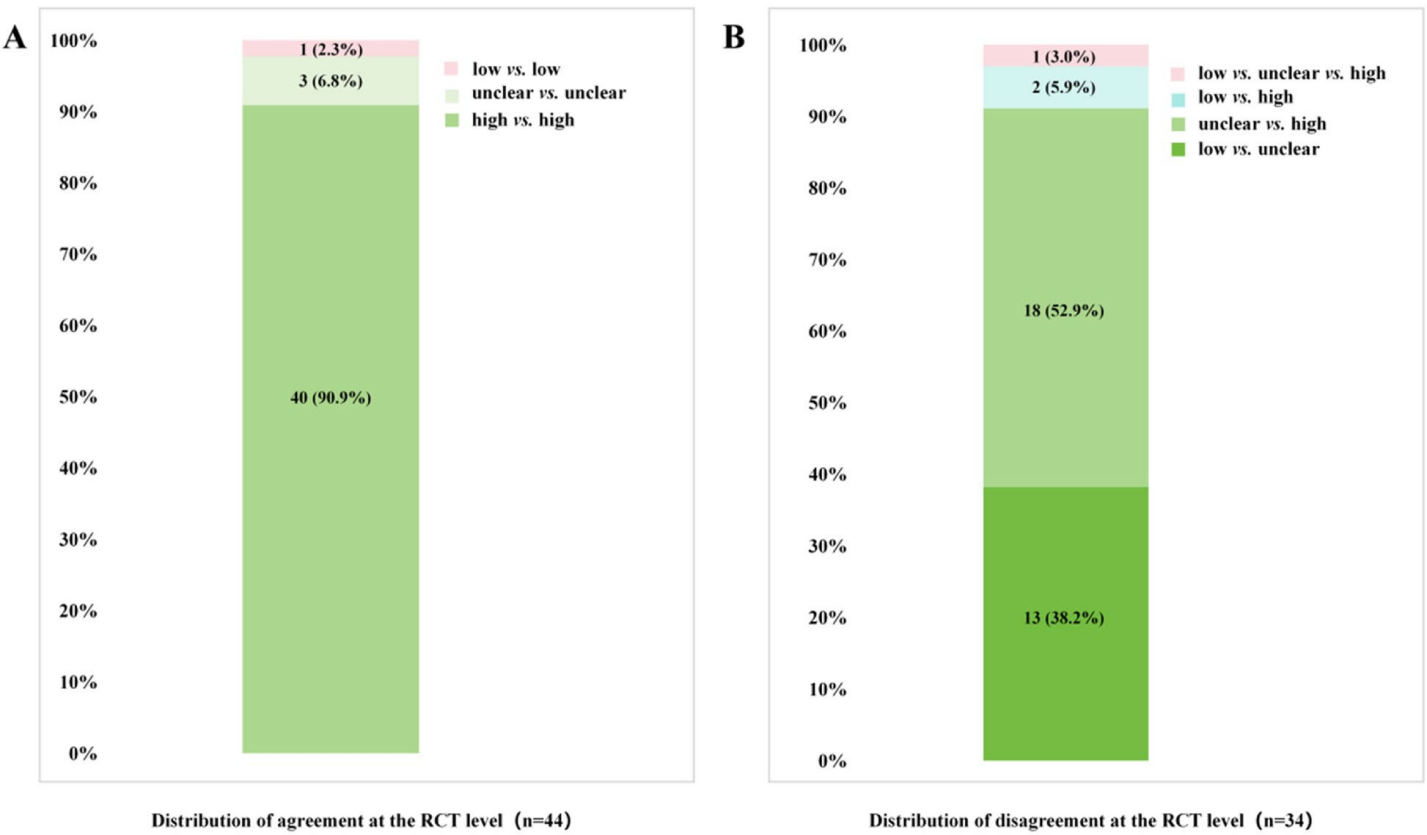
The distribution of agreement and disagreement of the RoB assessment at the RCT level. A: The distribution of agreement of the RoB assessment at the RCT level. B: The distribution of disagreement of the RoB assessment at the RCT leve

The assessment results were agreement in 44 (56.4%) RCTs and disagreement in 34 (43.6%) RCTs at the trial level. In the agreement group, high risk *vs.* high risk accounted for 90.9%. In the disagreement group, unclear risk *vs.* high risk and low risk *vs.* unclear risk accounted for 52.9% and 38.2%, respectively. The distribution of agreement and disagreement of the RoB assessment at the RCT level is shown in Fig. 3.

#### Assessment of each domain in the RoB

**Fig. 4:**
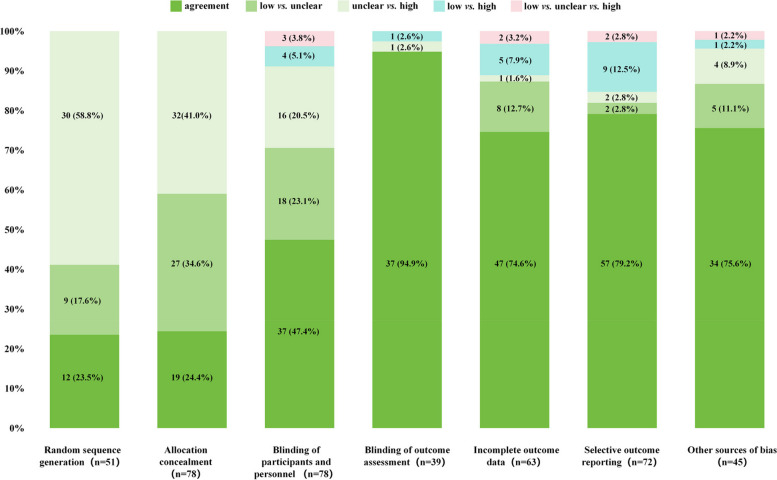
Distribution of agreement and disagreement for different RoB domains

“Random sequence generation” was assessed in 51 RCTs, and the assessment results were agreement in 12(23.5%) RCTs and disagreement in 39(76.5%) RCTs. In the disagreement group, there were 9 (17.6%) low risk *vs.* unclear risk and 30 (58.8%) unclear risk *vs.* high risk (Fig. 4).

“Allocation concealment” was assessed in 78 RCTs, of which 19 (24.4%) RCTs showed agreement of the RoB assessment. The disagreements included 27 (34.6%) low risk *vs.* unclear risk and 32 (41.0%) unclear risk *vs.* high risk (Fig. 4).

“Blinding of participants and personnel” was assessed in 78 RCTs, and the assessment results were agreement in 37 (47.4%) RCTs and disagreement in 41 (52.6%) RCTs. In the disagreement group, there were 18 (23.1%) low risk *vs.* unclear risk, 16 (20.5%) unclear risk *vs.* high risk, 4 (5.1%) low risk *vs.* high risk, and 3 (3.8%) low risk *vs.* unclear risk *vs.* high risk (Fig. 4).

“Blinding of outcome assessment” was assessed in 39 of RCTs, and the assessment results were agreement in 37 (94.9%) RCTs and disagreement in 2 (5.1%) RCTs. In the disagreement group, there were 1 (2.6%) unclear risk *vs.* high risk and 1 (2.6%) low risk *vs.* high risk (Fig. 4).

“Incomplete outcome data” were assessed in 63 RCTs that focused on the same outcomes in different reviews. The assessment results were agreement in 47 (74.6%) RCTs and disagreement in 16 (25.4%) RCTs. In the disagreement group, there were 8 (12.7%) low risk *vs.* unclear risk, 1 (1.6%) unclear risk *vs.* high risk, 5 (7.9%) low risk *vs.* high risk, and 2 (3.2%) low risk *vs.* unclear risk *vs.* high risk (Fig. 4).

“Selective outcome reporting” was assessed in 72 RCTs, and the assessment results were agreement in 57 (79.2%) RCTs and disagreement in 15 (20.8%) RCTs. In the disagreement group, there were 2 (2.8%) low risk *vs.* unclear risk, 2 (2.8%) unclear risk *vs.* high risk, 9 (12.5%) low risk *vs.* high risk, and 2 (2.8%) low risk *vs.* unclear risk *vs.* high risk (Fig. 4).

“Other sources of bias” were assessed in 45 RCTs, and the assessment results were agreement in 34 (75.6%) RCTs and disagreement in 11 (24.4%) RCTs. In the disagreement group, there were 5 (11.1%) low *vs.* unclear, 4 (8.9%) unclear *vs.* high, 1 (2.2%) low *vs.* high and 1 (2.2%) low *vs.* unclear *vs.* high (Fig. 4).

### Possible reasons for disagreement in the RoB assessment

At the trial level, there was no significant difference in the proportion of the year of publication ≤ 1996 and impact factor between the agreement and disagreement groups. At the domain level, the “allocation concealment” and “blinding of participants and personnel” had higher proportion of publication year ≤ 1996 in the agreement group (*P* = 0.008 and *P* < 0.001, respectively). For the “blinding of participants and personnel”, the impact factor was higher in the agreement group (*P* < 0.001) (Table 3). We analyzed the support text, and found that the most common reason for disagreement was related to extracting different information in the article. The other reason was that the reviewers considered differently in same or similar text, 41.0% for “random sequence generation”, 30.5% for “allocation concealment”, 29.3% for “blinding of participants and personnel”, 50.0% for “blinding of outcome assessment”, 6.7% for “selective outcome reporting”, and 9.1% for “other sources of bias”. The main reasons for differences in support text for each domain are reported in Table 4.

**Table 3:** The year of publication and impact factor of the journal between the agreement and disagreement groups for the RoB assessment

Domains	Agreement	Disagreement	*P* value
Risk of bias assessment at the RCT level (*n*)	44	34	
Impact factor [M(IQR)]	3.92(2.57, 27.81)	4.17(2.58, 23.60)	0.628
The year of publication ≤ 1996 [*n* (%)]	19(43.2)	14(41.2)	0.859
Random sequence generation (*n*)	12	39	
Impact factor [M(IQR)]	4.02(2.65,19.50)	2.64(0.69, 4.15)	0.095
The year of publication ≤ 1996 [*n* (%)]	6(50.0)	8(20.5)	0.103
Allocation concealment (*n*)	19	59	
Impact factor [M(IQR)]	7.19(3.70, 45.54)	3.97(2.32, 7.71)	0.052
The year of publication ≤ 1996 [*n* (%)]	13(68.4)	20(33.9)	0.008
Blinding of participants and personnel (*n*)	37	41	
Impact factor [M(IQR)]	7.71(4.17, 52.97)	2.64(1.24, 4.06)	< 0.001
The year of publication ≤ 1996 [*n* (%)]	26(70.3)	7(17.1)	< 0.001
Blinding of outcome assessment (*n*)	37	2	
Impact factor [M(IQR)]	7.71(4.17, 45.54)	32.05(3.70, 60.40)	0.897
The year of publication ≤ 1996 [*n* (%)]	27(73.0)	1(50.0)	0.490*
Incomplete outcome data (*n*)	47	16	
Impact factor [M(IQR)]	3.97(2.27,7.71)	13.89(5.65, 52.85)	0.148
The year of publication ≤ 1996 [*n* (%)]	17(36.2)	10(62.5)	0.066
Selective outcome reporting (*n*)	57	15	
Impact factor [M(IQR)]	4.17(2.45, 18.96)	6.11(2.64, 30.22)	0.428
The year of publication ≤ 1996 [*n* (%)]	26(45.6)	7(46.7)	0.942
Other sources of bias (*n*)	34	11	
Impact factor [M(IQR)]	2.58(0.98, 3.77)	23.60(6.94, 60.39)	0.001
The year of publication ≤ 1996 [*n* (%)]	4(11.8)	5(45.5)	0.046

*M* median, *IQR* interquartile range

^*^Fisher's exact test

**Table 4:** Main reasons for disagreements in assessment domains for RoB

RoB domains	Main reasons for disagreement	*n* (%)	Example
Random sequence generation	Considered differently in same or similar information in article	16(41.0)	-Support text: Quote: "Placed at random into treated (50) or control (49) groups. The two groups matched reasonably closely with regard to numbers, age, sex, and severity of hypertension." Comment: Method of randomization was not described. Probably randomization achieved as groups matched at baseline. Assessment of RoB: low risk -Support text: Quote: "placed at random into treated (50) or control (49) groups. The two groups matched reasonably closely with regard to numbers, age, sex, and severity of hypertension". Comment: method of randomisation was not described. Assessment of RoB: unclear risk
	Extract different information in article	21(53.8)	-Support text: Judgement Comment: Controlled before‐and‐after design. Assessment of RoB: high risk -Support text: Atorvastatin 10 mg/d; intervention was analysed, and as no placebo group was included for comparison, assessment of random sequence generation is not applicable. Assessment of RoB: unclear risk
	Consider differently by incomplete or unclear description	2(5.1)	-Support text: Quote: "Patients were randomly allocated". Assessment of RoB: low risk -Support text: Comment: probably done. Method of randomisation and allocation was not described; no other information is provided. Assessment of RoB: unclear risk
Allocation concealment	Considered differently in same or similar information in article	18(30.5)	-Support text: Allocation of individuals within matched pairs to treatment and control groups was made by a blinded statistical co‐ordinator; thought to be randomized, but not entirely clear (unpublished information as per personal conversation with author by Mulrow 1998). Assessment of RoB: low risk -Support text: Allocation of individuals within matched pairs to treatment and control groups made by a blinded statistical co‐ordinator; thought to be randomised but not entirely clear (unpublished information as per personal conversation with author from Mulrow 1998). Assessment of RoB: unclear risk
	Extract different information in article	18(30.5)	-Support text: Atorvastatin 10 mg/d; intervention was analysed, and as no placebo group was included for comparison, assessment of allocation concealment is not applicable. Assessment of RoB: unclear risk -Support text: Controlled before and after design. Assessment of RoB: high risk
	Consider differently by incomplete or unclear description	23(39.0)	-Support text: Insufficient information. Assessment of RoB: unclear risk -Support text: Adequate, use of Slow Sodium and placebo tablets. Assessment of RoB: low risk
Blinding of participants and personnel	Considered differently in same or similar information in article	12(29.3)	-Support text: Double‐blinded. Assessment of RoB: low risk -Support text: "…[a] randomised, double‐blind, parallel‐group, active‐controlled, dose‐titration study was performed…" (line 1 under "Study Design" p.418). No further information was given. Assessment of RoB: unclear risk
	Extract different information in article	28(68.3)	-Support text: Lipid parameter measurements unlikely influenced by lack of blinding. Assessment of RoB: low risk -Support text: Atorvastatin 20 mg/d; intervention was analysed, and as no placebo group was included for comparison, blinding status is not applicable. Assessment of RoB: unclear risk
	Consider differently by incomplete or unclear description	1(2.4)	-Support text: Placebo was used. Assessment of RoB: low risk -Support text: No information. Assessment of RoB: unclear
Blinding of outcome assessment	Considered differently in same or similar information in article	1(50.0)	-Support text: Lipid parameters were measured in a remote laboratory;No comparison possible. Assessment of RoB: LDL‐C:low risk;WDAE:high risk -Support text: Lipid parameters were measured in a remote laboratory. No comparison possible Assessment of RoB: LDL‐C:low risk;WDAE:unclear risk
	Extract different information in article	1(50.0)	-Support text: Use of random zero sphygmomanometer. Assessment of RoB: low risk -Support text: Open study: Detection bias due to knowledge of the allocated interventions by outcome assessors. Assessment of RoB: high risk
Incomplete outcome data	Considered differently in same or similar information in article	2(12.5)	-Support text: 5/42 (11.9%) of the rosuvastatin group were not included in the efficacy analysis due to dropout or incomplete evaluation. Assessment of RoB: high risk -Support text: 2/42 (4.8%) participants were not included in the efficacy analysis. Assessment of RoB: low risk
	Extract different information in article	9(56.3)	-Support text: All participants who were randomised completed the study. Assessment of RoB: low risk -Support text: Lost to follow up, LS: 2/19; US: 0/19. Assessment of RoB: unclear risk
	Consider differently by incomplete or unclear description	5(31.3)	-Support text: Did not report dropout rate. Assessment of RoB: unclear risk -Support text: SBP, DBP, HR reported without SD or SEM. Assessment of RoB: high risk
Selective outcome reporting	Considered differently in same or similar information in article	1(6.7)	-Support text: Protocol not available to confirm reporting bias. Mortality rate and recurrence rate of strokes mentioned as study objectives were reported in results section. Quote: "Figures for minor strokes or transient cerebral ischaemic attacks are not available." Assessment of RoB: low risk -Support text: Protocol is not available to confirm reporting bias. Mortality rate and recurrence rate of strokes mentioned, as study objectives were reported in the results section."Figures for minor strokes or transient cerebral ischaemic attacks are not available". Assessment of RoB: unclear risk
	Extract different information in article	13(86.7)	-Support text: HR were not reported. Assessment of RoB: high risk -Support text: All outcomes reported. Assessment of RoB: low risk
	Consider differently by incomplete or unclear description	1(6.7)	-Support text: The ACCORD investigators elected to restrict analysis and reporting of SAE data to events related to blood pressure medications because those were the only events collected in a consistent manner throughout the trial and subject to safety officer and DSMB review. Assessment of RoB: high risk - Support text: 0. Assessment of RoB: low risk
Other sources of bias	Considered differently in same or similar information in article	1(9.1)	-Support text: "Part of the expenses of this research project was covered by a grant from the clinical research subcommittee of the North West Metropolitan Regional Hospital Board." Assessment of RoB: Low risk -Support text: "Part of the expenses of this research project was covered by a grant from the clinical research subcommittee of the North West Metropolitan Regional Hospital Board." Assessment of RoB: low risk -Support text: Industry sponsorship bias: "Part of the expenses of this research project was covered by a grant from the clinical research subcommittee of the North West Metropolitan Regional Hospital Board". Assessment of RoB: unclear risk
	Extract different information in article	10(90.9)	-Support text: source of funding not reported. Assessment of RoB: unclear risk -Support text: AstraZeneca funded the study; data may support bias against atorvastatin. Assessment of RoB: high risk

## Discussion

In this study, the level of agreement and disagreements in RoB assessments for RCTs included in more than one hypertension-related Cochrane review was explored. The level of agreement varied from domain to domain. “Blinding of outcome assessment” showed a reasonably high level of agreement (94.9%), and “incomplete outcome data”, “selective outcome reporting” and “other sources of bias” showed a moderate level of agreement. However, the domains of “random sequence generation” and “blinding of participants and personnel” showed low levels of agreement (24.4% and 47.4%, respectively). The agreement of “allocation concealment” was the worst, accounting for only 23.5%. This study revealed that there was a significant amount of disagreement in the RoB assessments among Cochrane reviews in the field of hypertension.

### Comparison with other studies

Jordan et al. [15] assessed the agreement in RoB judgments across 34 reviews for 46 RCTs that appeared in more than one Cochrane review of subfertility. They found that RoB assessments disagreed in 29% ~ 65% of domains, with the domain of “blinding of outcome assessment” showing the highest disagreement. In contrast, the current results indicated that the domain of “blinding of outcome assessment” had the lowest disagreement in the field of hypertension. The disagreements in the RoB assessments were related to the research field, and the reasons were needed to be further explored. Bertizzolo et al. [14] reported that the assessment results of “random sequence generation” and “allocation concealment” were highly consistent (81% *vs.* 74%), and the most inconsistent assessment was for “incomplete outcome data” (43%). This study revealed that the agreement of “random sequence generation” and “allocation concealment” was poor. The disagreement might be related to the publication date of RCTs. It was found that the agreement group had higher proportion of publication year ≤ 1996 than the disagreement group (*P* = 0.008) in the “allocation concealment”. A similar trend was found for the “random sequence generation”, although not statistically significant. We propose a conjecture that RCTs published before 1996 did not refer to the reporting guideline of CONSORT statement, which might have led to a lack of description of key domains such as “random sequence generation” and “allocation concealment”. Reviewers might tend to give unclear or high risk judgments, which would increase the agreement of the RoB assessment. The RCTs published after 1996 were generally referred to the CONSORT statement. In that process, because the scale of some domains was not the same, the content reported might not be the same. It was difficult for the reviewers to control the scale when assessing the content of these reports, which might lead to a higher possibility of disagreement. For example, the proportion of “high risk *vs.* unclear risks” was relatively high. However, papers published after 1996 are not necessarily guided by the CONSORT statement. Therefore, further research is needed. Previous study did not consider the impact of different outcomes on “incomplete outcome data” [14]. However, different reviews differed in the assessment of “incomplete outcome data” because they focused on different outcomes. In the study, we only analyzed on the domain “incomplete outcome data” of RCTs that focused on the same outcomes from different reviews to avoid the limitations. Only 54.5% of trials assessed the domain of “other bias” in the study, and previous studies did not consider this domain because it was difficult to assess “other sources of  bias” [14]. Babic et al. reported that Cochrane authors mention a wide range of sources of “other sources of  bias” in the RoB tool [19]. The Revised Cochrane RoB tool (ROB 2.0) deleted this domain to overcome some limitations of the “other sources of  bias” included in the original version [20].

The assessment of RoB was subjective, and it was possible that the reviewers were not using the same definitions for assessing RoB in some domains or that they did not interpret the evidence in the same way. Thus, the Cochrane handbook recommended that at least two independent reviewers assess the RoB, and differences should be resolved through discussion or a third reviewer. The RoB of an RCT is best assessed by reviewers with a high level of training and experience. In the current study, only Cochrane reviews were included. For Cochrane reviews, due to the high standards and stringent requirements, reviewers might have higher expedience. If researchers want to do a Cochrane review, there are several requirements for the researchers, such as at least 1 researcher who has completed a Cochrane review and at least 1 methodological expert in the team. Thus, there might be low variation in the authors' experience and understanding of the domains. However, in this study, we found a large number of disagreements for the same RCT, and different reviewers extracted different information for the same RoB domains. Thus, we should further explore this factor in future. In addition, it suggested building a standardized database of RoB based on the assessment by qualified reviewers to reduce the impact of reviewers’ experience. Standardized training for RoB assessment was also important [21]. To improve agreement, a study updated the RoB tool in 2019 (RoB 2.0), and RoB 2.0 refined the evaluation process in each field, combined “random sequence generation” and “allocation concealment” into bias in the randomization process, removed “other biases”, and used an example for clear instructions [20]. However, one study showed that the RoB 2.0 also showed low inter-rater reliability, and intensive training is needed before its application to improve reliability [22]. This study suggested that there is still room for improvement in the quality of RoB assessments. It would be helpful to have more training for reviewers on the assessment of RoB.

The characteristics of the RCTs included in the systematic reviews might also influence the RoB assessment. Therefore, the characteristics of the included RCTs were analyzed. One-third of RCTs were published in top journals, such as the *Lancet*, *BMJ*, *JAMA* and *Hypertension*. The median impact factor (IF) for journals was 4.2 (2.6, 23.6). It showed that journals that published RCTs in the field of hypertension were uneven. Previous study have found a correlation between the IF of journals that published RCTs and RoB assessment scores [23]. High impact factor journals required higher quality RCTs and more standardized reporting in general. In our study, we also found that the impact factor was higher in the agreement group than disagreement group (*P* < 0.001) in the “blinding of participants and personnel”. Thus, it might be one of the possible reasons for disagreement for RoB assessment. In addition, it is worth noting that over half of the RCTs included in our analysis were initiated prior to 2005. It is important to mention that since 2005, the International Committee of Medical Journal Editors implemented a policy requiring the registration of clinical trials [24]. This policy has been instrumental in reducing reporting bias [25] and enhancing the overall quality of hypertension studies. Therefore, more rigorous quality control measures are needed to ensure that high-quality RCTs are included in Cochrane reviews.

### Contributions and implications

This study defined disagreements of RoB assessment for RCTs included in three or more reviews by adding a low *vs.* unclear *vs.* high category. Second, the authors performed a more comprehensive analysis of 7 RoB domains compared with only 5 domains analysed in previous studies [14, 26].

### Limitations

The study had several limitations. First, when an RCT included by three or more systematic reviews, the same judgments were made across multiple reviews, which were combined so that only discrepancies were highlighted as a proportion. As a result, it might be difficult to accurately see the level of agreement and disagreement separately between reviews. Second, this study only preliminarily studied those factors, leading to disagreement of the RoB assessment. In the future, more studies are needed to explore these factors. Third, the study evaluated the reliability of the 2011 version of the RoB tool only, not the RoB 2.0. Therefore, the effectiveness of our study findings is limited to the the 2011 version of the RoB tool.

## Conclusions

For Cochrane reviews in the field of hypertension using the 2011 version of the RoB tool, there was large disagreement in RoB assessment. It is suggested that the RoB assessments in systematic reviews that used the 2011 version of the RoB tool be interpreted with caution. More accurate information from RCTs needs to be collected when we synthesize clinical evidence.

### Supplementary Information

Supplementary Material 1: Details of journals published by included RCTs.

Supplementary Material 2: The data set for this study.

Supplementary Material 3: The STROBE checklist of the article.

## Abbreviations

RCTs: Randomized controlled trials
RoB: Risk of bias
