# Supplementary material for: Disagreements in risk of bias assessment for randomized controlled trials in hypertension-related Cochrane reviews
Source: Trials. 2024 Jun 21;25:405. doi: 10.1186/s13063-024-08145-2 (PMC11191165; doi:10.1186/s13063-024-08145-2)
Supplement: Supplementary file 1 — Supplementary Material 1: Details of journals published by included RCTs. [file 13063_2024_8145_MOESM1_ESM.docx]

Details of journals published by included RCTs.

| Journal | Number of RCTs |
| --- | --- |
| Lancet | 9 |
| Journal of Hypertension | 7 |
| BMJ | 6 |
| Hypertension | 6 |
| Clinical Therapeutics | 2 |
| European Journal of Clinical Pharmacology | 2 |
| JAMA | 2 |
| Therapeutic Research | 2 |
| Advances in Therapy | 1 |
| American Heart Journal | 1 |
| American Journal of Hypertension | 1 |
| Annals of Vascular Diseases | 1 |
| Archives of Internal Medicine | 1 |
| Archives of Sexual Behavior | 1 |
| British Journal of Clinical Pharmacology | 1 |
| British Journal of Clinical Practice | 1 |
| Chinese Journal of New Drugs and Clinical Remedies | 1 |
| Circulation | 1 |
| Clinical & Experimental Medicine | 1 |
| Current Medical Research & Opinion | 1 |
| Current Therapeutic Research Clinical and Experimental | 1 |
| Diabetic Medicine: a Journal of the British Diabetic Association | 1 |
| Diabetology International | 1 |
| European Journal of Clinical Investigation | 1 |
| Guangdong Yixue | 1 |
| Japanese Heart Journal | 1 |
| Journal of Atherosclerosis & Thrombosis | 1 |
| Journal of Cardiovascular Pharmacology | 1 |
| Journal of Cardiovascular Pharmacology and Therapeutics | 1 |
| Journal of Clinical Pharmacology | 1 |
| Journal of Family Practice | 1 |
| Journal of the American College of Cardiology | 1 |
| The Medical Journal of Australia | 1 |
| New England Journal of Medicine | 1 |
| Nutrition Metabolism & Cardiovascular Diseases | 1 |
| Scandinavian Journal of Clinical and Laboratory Investigation | 1 |
| The Annals of Pharmacotherapy | 1 |
| Zhongguo Xinyao yu Linchuang Zazhi | 1 |
